# Beyond the Bench: Continuing Education for Nurses on Environmental Genetics and Complex Diseases

**DOI:** 10.1289/ehp.114-a410

**Published:** 2006-07

**Authors:** Tanya Tillett

Many people find it hard to fit professional development and continuing education into their busy work lives. Now help is just a mouse-click away for nurses seeking flexible, self-paced training in the growing field of environmental genetics. The Community Outreach and Education Core (COEC) of the Center for Environmental Genetics at the University of Cincinnati, in collaboration with the Genetics Education Program for Nurses (GEPN) of Cincinnati Children’s Hospital Medical Center, has created an online Environmental Genetics and Complex Diseases educational module that introduces nurses to the principles of environmental genetics, and also teaches them how to apply those principles in nursing practice.

Online since December 2005, the module is useful for all nurses in clinical practice, but especially targets those who work extensively with minority or medically underserved patients. The module focuses on alcoholism, lead, and asthma, three challenging public and environmental health problems in underserved communities.

“The module is designed to prepare nurses in underserved communities to identify people who are at risk for environmental genetic conditions and help those people gain access to community services that emphasize prevention and early treatment strategies,” says COEC director M. Kathryn Brown.

Cynthia Prows, a clinical nurse specialist in genetics and the principal investigator of the web program, says the module organizes information into useful and manageable resources. “There is a tremendous amount of information on the Internet about genetics and about environmental health. But how do nurses who have limited knowledge in the topic areas locate the various sites, sift through all the information, decide what information is current and accurate, and then use that information for learning purposes? The answer is, most nurses don’t because they don’t have the time or the necessary foundational knowledge in genetics to mine the overwhelming mass of information that is accessible through the Internet.”

The module developers have done that work for the nurses, and have organized the content in a way that helps nurses develop foundational knowledge in environmental genetics using high-quality resources that are applicable to their practice. Once learners create a unique username and password, they can access the module free of charge, and can re-enter it at any time at the place they last exited. Those who wish to earn 4.8 nursing continuing education contact hours after completing the module and associated evaluations pay a minimal processing fee.

The module offers nurses background information on gene–environment interactions, and teaches them environmental and sociodemographic risk factors for common diseases. It also provides screening tools and community resources for nurses treating patients with recognizable genetic and environmental risk factors. Each of the three learning tracks also offer prenatal, pediatric, and adult case studies and self-assessments with each content area.

After completing the module, nurses are able to approach their communities armed with valuable knowledge of gene–environment interactions and insight into how those interactions can affect human health. They are also equipped with a wealth of online resources that can be accessed long after they complete the training module.

“Making sense of the fast-growing literature about how the health impacts of environmental exposures through the life span are mediated by our genetics is a challenge for health care professionals,” says Brown. “We hope that the vast array of resources identified in these self-paced, online modules will be helpful to primary care practitioners trying to make sense of new developments in genetic screening tests, environmental prevention strategies, and treatment options.”

The module is available at http://gepn.cchmc.org/. Three additional genetics education modules currently available include Promoting Informed Decision-Making about Genetic Testing, Ethical and Social Issues Related to Genetic Testing, and Interpreting Family History. Two new modules are also in the pilot testing phase: Genetics Is Relevant Now––Nurse Views and Patient Stories, and Nurses’ Role in Pharmacogenetics/Pharmacogenomics.

## Figures and Tables

**Figure:**
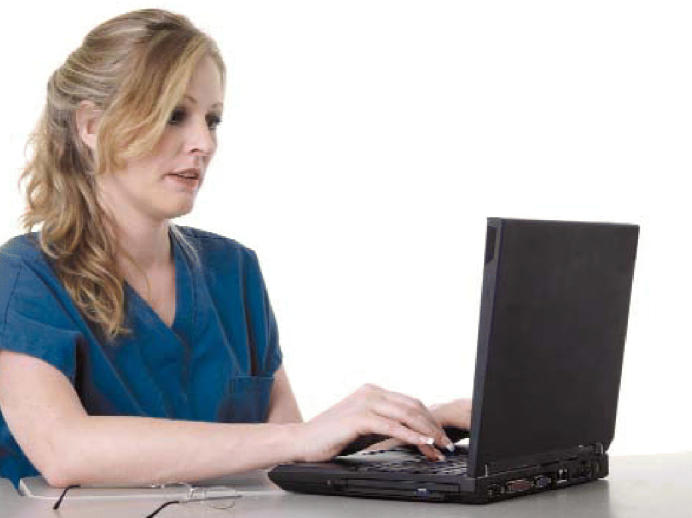
New age of nursing An online continuing education module introduces nurses to principles of environmental genetics and shows them how to put such principles into practice.

